# Laparoscopic Management of Heterotopic Interstitial Pregnancy with Subsequent Term Delivery 

**DOI:** 10.22074/ijfs.2015.4250

**Published:** 2015-07-27

**Authors:** Yong-Soon Kwon, Sang-Hun Lee, Kyong Shil Im, Jae Hun Ro

**Affiliations:** 1 Department of Obstetrics and Gynecology, College of Medicine, University of Ulsan, Ulsan University Hospital, Ulsan, Korea; 2 Department of Anesthesiology and Pain Medicine, College of Medicine, Catholic University of Korea, Uijeongbu St Mary’s Hospital, Uijeungbu-City, Korea

**Keywords:** Cesarean Delivery, Heterotopic Interstitial Pregnancy, Laparoscopic Surgery

## Abstract

A 35 year-old woman at 7-week gestational age was referred to our hospital. The patient was diagnosed with the heterotopic interstitial pregnancy by transvaginal ultrasonogra- phy after receiving *in vitro* fertilization (IVF) and embryo transfer. Laparoscopic excision and curettage was successfully performed at 8.4-gestational age under general anesthesia and the patient was discharged 2 days after operation without any post-operative complications. The woman had normal antenatal follow-up and deliv- ered a healthy baby at term by cesarean section.

## Introduction

Heterotopic pregnancies, where intrauterine and ectopic gestations co-exist, are very rare with an estimated incidence of 1 in 30,000 pregnancies (1). However, this may be as high as 1% in the setting of *in vitro* fertilization (IVF) where multiple embryos are transferred (2). Recently, there is an increased incidence of abnormal pregnancies such as heterotopic pregnancy because the number of women exposed to risks, such as pelvic inflammatory disease, previous pelvic surgery, tumors, uterine anomalies, and the use of assisted reproductive technologies (ART) increases.

There are limited options for the treatment of heterotopic pregnancy, particularly if the woman desires to continue with her intrauterine pregnancy.

We reported a case of successful laparoscopic surgery for heterotopic interstitial pregnancy and subsequent successful delivery at term.

## Case Report

**Fig.1:**
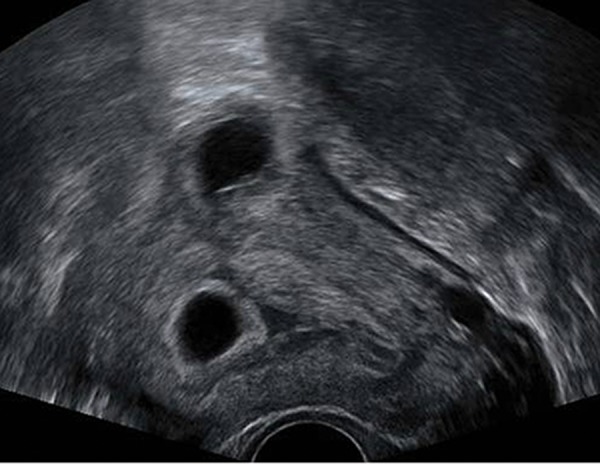
Transvaginal ultrasonography showed the gestational sacs in the two different sites, longitudinal and sagittal views.

A 35-year-old woman was referred to our department for the further management of a heterotopic interstitial pregnancy. This heterotopic pregnancy was the first episode of pregnancy after the patient received an IVF for primary infertility due to previous surgeries which included right salpingectomy and left tubal pregnancy, respectively. The patient presented with acute abdominal pain, localized to the left lower quadrant area with local tenderness on physical examination. Transvaginal ultrasonography showed an intrauterine pregnancy of about 7 weeks gestation and another gestation of about 4.8×4.5 cm at the left interstitial of the fallopian tube (Fig.1).

**Fig.2:**
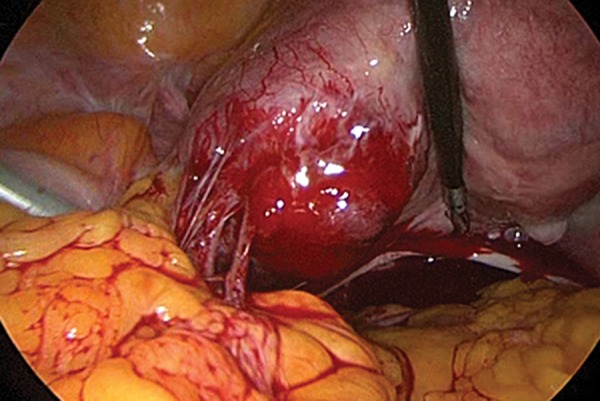
A laparoscopic view showed the mass located in the left cornus of the uterus. The cornus was expanded and had a bulging appearance.

Laparoscopic surgery was performed at 8 weeks gestational age. The intra-abdominal pressure was maintained at 13 mmHg with carbon dioxide gas. Once the pneumoperitoneum was achieved, video- laparoscopy (laparoscopic camera provided by Stortz, Germany) was performed using a 10-mm trocar that had been introduced through the umbilicus. Further, three trocars were needed for the operation. A 10-mm trocar for placement of the endoscopic suturing was placed on the left side, a 5-mm trocar on the right side of the lower abdomen, and another 5-mm trocar on the median line just above the pubic hairline. The left cornus of the uterus was distended with increased vascularity (Fig.2). The ipsilateral ovary and fallopian tube were grossly normal in appearance.

**Fig.3:**
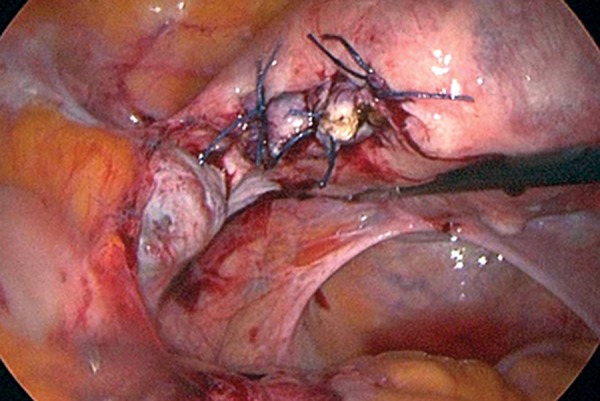
The technique of laparoscopic suturing is a simple interrupted suturing. The ipsilateral ovary was grossly normal in appearance.

The bulging cornus was transversely incised using a monopolar cutting electrode (The ENDOPATH ® Electrosurgery, EPH02, Ethicon inc.) 40 W to expose the ectopic gestation. The mass was removed followed by curettage of the area with a spoon forceps to completely evacuate the ectopic gestation. Laparoscopic suturing was done using 1-0 POLYSORB™ sutures polysorb (Covidien inc.) after controlling the bleeding on the surgical bed of the left cornus. The technique of laparoscopic suturing was a simple interrupted suturing with caution taken to avoid damage to the intrauterine pregnancy (Fig.3).

The time of operation was 40 minutes and the time of anesthesia was 55 minutes. There was no intraoperative complication. A closed drain bag was inserted during the operation. The patient was discharged 2 days after operation without any postoperative complications. During the time from initiation of antenatal care to delivery, the patient stayed healthy and showed no clinical problems. The placenta was located centrally in anterior body of the uterus. Finally, an elective cesarean delivery was performed at 38 weeks gestation and a 3.4 kg healthy female baby was born. Minimal old scars without major deformity or adhesion were found on the cornual operation site.

## Discussion

Heterotopic pregnancy is believed to occur in 1% of all conceptions achieved with IVF, but implantations, specifically in the interstitial, account for only 1% of all ectopic pregnancies (3). There is little agreement regarding the optimal surgical management of heterotopic pregnancy because of the rarity of this type of pregnancy.

To our knowledge, there have been six reports of laparoscopic management of interstitial heterotopic pregnancies (2, 4-8). Two of the six cases with laparoscopic management of intrauterine pregnancy were reported without a good outcome.

In the current case, there were no complications during the laparoscopic operation and no late miscarriage. An elective cesarean delivery was done and a healthy 3.4 kg female baby was born at 38-weeks gestational age.

A selective embryo reduction by direct local injection of potassium chloride or hyperosmolar glucose solution could be considered in women with clinically stable situation. However, the patient in the current case presented with severe abdominal pain and hemoperitoneum with a risk of catastrophic rupture of the interstitial pregnancy; therefore, surgical management was recommended.

The minimal invasive laparoscopic surgery for the interstitial pregnancy was focused on the prevention of intraoperative complications: i. operation time and anesthetic time had to be shortened, ii. the amount of blood loss had to be reduced and iii. during suturing, the tip of the needle must have some distance from the normal gestational sac in the intrauterine cavity.

We did not use pharmacological methods such as vasopressin to control bleeding in order to avoid any potential effect on the circulation to the normal intrauterine. Instead, we tried to shorten the time between the staring time of incision and finishing of suturing to reduce the amount of intraoperative bleeding loss.

Laparoscopic suturing technique is a key factor to reduce the operation time and bleeding. With regard to these points, an expert laparoscopic surgeon can help to carry out a laparoscopic surgical management for a heterotopic pregnancy with less intraoperative bleeding.

Although the increased rates in pregnancy after IVF represent a welcomed trend in the advanced reproductive technologies, the gains have not eliminated the risk of an ectopic pregnancy (9). In fact an iatrogenic transfer of multiple embryos to the uterus after an IVF represents a major risk factor for a heterotopic pregnancy (10).

We suggest that the aim of treatment for heterotopic pregnancy should be the continuation of the normal pregnancy and the careful use of minimal invasive surgical techniques for the ectopic pregnancy. Expert laparoscopic management for a heterotopic pregnancy might be the appropriate treatment modality for fetal and maternal safety. A collaborative approach for larger collection of data on these surgical techniques could help women with heterotopic pregnancy continue their normal intrauterine pregnancy safely.
